# Microplastics in Different Tissues of a Commonly Consumed Fish, *Scomberomorus guttatus,* from a Large Subtropical Estuary: Accumulation, Characterization, and Contamination Assessment

**DOI:** 10.3390/biology12111422

**Published:** 2023-11-12

**Authors:** Mohammad Belal Hossain, Farjana Haque Pingki, Md. Abdus Samad Azad, As-Ad Ujjaman Nur, Partho Banik, Bilal Ahamad Paray, Takaomi Arai, Jimmy Yu

**Affiliations:** 1 School of Engineering and Built Environment, Griffith University, Brisbane, QLD 4111, Australia; 2 Department of Fisheries and Marine Science, Noakhali Science and Technology University, Noakhali 3814, Bangladesh; 3 Department of Applied Chemistry and Chemical Engineering, Noakhali Science and Technology University, Noakhali 3814, Bangladesh; 4 Department of Zoology, College of Science, King Saud University, P.O. Box 2455, Riyadh 11451, Saudi Arabia; 5 Environmental and Life Sciences Programme, Faculty of Science, University Brunei Darussalam, Jala Tungku Link, Gadong BE1410, Brunei Darussalam

**Keywords:** microplastics, contamination, king mackerel, polymers, estuarine fish, bioaccumulation, Bangladesh

## Abstract

**Simple Summary:**

It is evident that microplastics can enter the human body via dermal contact, inhalation, and food intake and pose a significant threat to human health. Therefore, understanding microplastics is essential for protecting the environment and human health. This study identified 48.7 MPs on average in each fish of king mackerel, with varying concentrations in different tissues, such as the digestive tract, gills, and muscle. The size and characteristics of these MPs varied but many were <0.5 mm in size (97.74%) and fiber-like, with a lot in the muscle tissue, which raises concerns for human consumption. Three types of plastic polymers were identified in the MPs, likely from things like food packaging and plastic waste. The fish’s muscle and digestive tract were significantly contaminated with MPs, indicating a high level of pollution.

**Abstract:**

Microplastics (MPs) ingestion by fish signifies a worldwide threat to human health but limited research has examined their existence within the consumable portions (muscle) of fish. Thus, this study was undertaken to unveil the prevalence, characterization, and contamination extent of MPs across various body tissues, including the muscle of the king mackerel (*S. guttatus*) from the lower Meghna estuary in Bangladesh—a pioneering investigation in this region. In our analysis, we identified a total of 487 MPs, with an average abundance of 48.7 ± 20.3 MPs/individual. These MPs were distributed across different tissues, with respective concentrations of 0.84 ± 0.45 items/g in the digestive tract, 2.56 ± 0.73 items/g in the gills, and 0.3 ± 1.72 items/g in the muscle tissue. The observed variations among these tissue types were statistically significant (*p* < 0.05). Moreover, a significant positive correlation indicated that fish with higher weight had higher MPs in their gills and DT (digestive tract). The majority were <0.5 mm in size (97.74%) and exhibited a fiber-like shape (97.74%), with a notable prevalence of transparent (25.87%) and a pink coloration (27.92%). Remarkably, the majority of MPs were discovered within the size range of <0.5–1 mm (100%), particularly in the muscle tissue, signifying a substantial transfer of MPs into the human diet. Besides, we discovered only three polymer types of microplastics which could be attributed to the extensive use of food packaging, plastic containers, wrapping plastics, residential garbage, and plastic pipes that end up in the aquatic environment via river discharges. The contamination factor (CF) values of fish muscle (5.75) and the digestive tract (5.50) indicated that these fish organs were considerably contaminated (3 < CF < 6) with MPs. The pollution index of MPs (PLI > 1) indicated a high contamination level for MPs pollution of *S. guttatus* in the lower Meghna River estuary.

## 1. Introduction 

Microplastics (MPs) (<5 mm in size), made of a synthetic polymeric matrix, significantly contribute to ocean plastic pollution by quantity due to their wide range of applications, including in medical devices, electrical safety materials, clothing and textiles, fisheries equipment, packaging, thermal insulation, and solid and water soluble particles [1,2,3]. More than five trillion plastic particles, totaling over 250,000 tons in mass, have been estimated to be floating in the surface ocean alone where more than 90% of these particles are classified as MPs [4]. The amounts are increasing every year with the increased population size globally and mismanagement of plastic waste. These plastics or MPs pose a global threat to aquatic ecosystems and animal and human health because they contain a variety of chemical additives, including polythene (PE), polypropylene (PP), polythene terephthalate (PET), polystyrene (PS), and polyvinyl chloride (PVC) [5]. They also act as carriers of pathogens that cause disease, heavy metals, and other life-threatening toxins [6]. 

Based on sources, MPs are categorized into two types: primary MPs (granules, pellets, and microspheres) manufactured for use in cleaning, personal care, and cosmetic products and secondary MPs, created when larger particles are broken down or fragmented by mechanical abrasion and photochemical oxidation in the environment and are thought to be readily bioavailable to organisms worldwide [7]. According to recent research, MPs can fragment in the environment, becoming progressively smaller and lower in density before finally forming nanoplastics (≤1 μm in size) [8].

A variety of organisms can ingest MPs since they are small and persistent in the ecosystem [9]. Fish, birds, invertebrates, and marine mammals have all been found to consume MPs [10,11]. Once MPs are consumed, they build up in body tissue and are likely to have adverse effects in fish, including decreased feeding and growth, low fecundity, and low survival rates [12]. In addition, fish, as sources of human food, have recently received significant attention because of the risks associated with the bioaccumulation of MPs and possible biomagnification for a variety of hydrocarbons, heavy metals, dyes, and other contaminants in it [13]. By realizing the facts, the detection of MPs in the gills and digestive tracts of fish in marine environments from neighboring countries, e.g., India and China, has received a great attention in earlier research [11,14,15,16]. In Bangladesh, despite several studies [17,18,19] that looked at the intake of plastics by various fish species from the marine environment, to our knowledge, there are no published documents detailing the consumption of MPs by king mackerel fish, *S. guttatus*. This migratory fish is found throughout the Indo-West Pacific, Bangladesh, India, and Sri Lanka as far as southeast Asia and is popularly eaten in Bangladesh [15,18]. Earlier studies did not focus on MPs levels in the muscle of any marine fish [15,18]. Given that the most edible part of fish is the muscle and that people are highly concerned about the potential risks of pollutants in muscles, it is imperative to examine the contaminants present in fish muscle [20]. Therefore, this study aimed to assess the prevalence, characterize and identify the polymer types of MPs in different body tissues including the muscle of *S. guttatus* fish, and assess their contamination level in a sub-tropical estuary of Bangladesh for the first time. The results will indicate the MPs contamination status of the Meghna Estuary and will be useful in determining any potential risks to human health from consuming this fish.

## 2. Materials and Methods

### 2.1. Study Area

The Meghna River Estuary, one of the largest, stretches for 160 km from Chandpur in the south to Tetulia. It is, however, spreading between the Tetulia and Shahbazpur rivers, both of which have a sea-face width of about 40 km. It has a noteworthy and varied ecosystem that contributes to the region’s socioeconomic well-being in a number of areas, including farmland, industrial use, drinking water sources for the millions of residents who live nearby, and fisheries. Fisheries’ resources serve as spawning, feeding, and nursery grounds for both freshwater and marine fish species; their availability changes according to the river’s water discharge volume and tidal range. The greatest effect on the environment in coastal zones around the globe comes from pollution caused by numerous small- and large-scale industrialization, modernized urbanization, and newly adopted farming techniques. Numerous types of agricultural waste, excrement and faces, oil spills from passenger and fishing vessels, and various minor industrial effluents are infiltrating estuarine systems which are home to enormous amounts of synthetic wastes like plastic and other wastes [21]. The highest concentrations of specific water parameters in comparison to the RPI index clearly show that the lower Meghna River Estuary has been chosen as a polluted estuary. 

### 2.2. Fish Sample Collection and Preparation

The king mackerel, locally known as Surma (*S. guttatus*), one of the most edible estuarine fish in Bangladesh, was selected for this study. Samples were taken using the estuarine set bag net (ESBN or Behundi jal) which was used in the lower Meghna river in shallow littoral waters between 2–3 m deep . Between March and April 2022, a total of 10 *S. guttatus* fish specimens were collected for this research. Then, the fish were kept in an icebox and transported to the laboratory of Coastal and Marine Science, Noakhali, where they were kept in a −20 °C refrigerator for further MP analysis. After the fish samples had thawed at room temperature in a laboratory container, blood, debris, and sediments were washed away using Mill-Q distilled water. A digital weighing scale (BSA224S, Sartorius, Shanghai, China) was used to weigh the body weight [22] and a measuring tap or ruler was used to assess the standard length (SL), total length (TL), and fork length (FL). Then, the fish specimens were dissected using sharp clean scissors. The dissection was performed in a clean and controlled environment such as a laboratory with laminar flow hoods to minimize the introduction of external contaminants. Personnel involved in the dissection should wear appropriate personal protective equipment, including gloves and lab coats. The whole digestive tract (DT), gills, and only 5 g of fish muscle tissue were removed from each specimen independently, weighed, and then transferred to a Petri dish in order to determine the concentration of MPs ingestion in fish [18,23]. To minimize the chance of contamination for peroxide digestion, all the fish tissues were transferred into a 1 L glass beaker and wrapped in aluminum foil after dissection [15].

### 2.3. Digestion of Fish Tissue and Separation of MPs

Hydrogen peroxide (H_2_O_2_, Scharlab, Barcelona, Spain) was used to digest fish tissue, including the gills, digestive system, and muscle, in a manner similar to that described by Karami et al. [23] with a few minor adjustments (e.g., omitting density separation by (1.2 g/mL) NaCl after digestion because of less amount of organic matter left). In order to digest biogenic material, 30% hydrogen peroxide (H_2_O_2_) was added at a ratio of 1:20 (*w*/*v*) into a 1 L glass beaker containing fish tissue separately. This method is more efficient, according to studies [24], than using sodium hydroxide (NaOH) or hydrochloric acid (HCl) [25,26]. Before moving on to the next stage, the entire acid-tissue mixtures were left on the lab bench at room temperature for a short while. The digestion combination was warmed on a magnetic hotplate stirrer to a temperature of 55 to 65 °C at a speed of 75 rpm until H_2_O_2_ was evaporated [25]. If the organic substance had not been completely digested by then, additional H_2_O_2_ (nearly 1 mL, 1–2 mL) was added [26,27]. Samples were transferred to a density separator for 24 h with a NaCl (1.2 g/mL) solution after all the tissue had been removed through digestion [28]. Then, 5.0 µm cellulose nitrate filter paper (Minipore, Ghaziabad, India) with a 47 mm width was used to filter the supernatant solution from the separator [4].

### 2.4. Microscopic Analysis and Polymer Identification of MPs 

A light stereomicroscope (Leica EZ4E, Leica Microsystems, Wetzlar, Germany) with 8× to 35× magnification was used to identify and quantify MP particles from the filter [29,30,31,32]. The MPs were counted individually on each quadrate of filter paper. We used a quadrant size of 47 mm × 47 mm (2209 square millimeters) for counting microplastics on filters. All microplastics found within that grid were counted individually. Once microplastics had been counted within the defined grid, the total number of microplastics on the entire filter was extrapolated. Then, the count of microplastics within the grid by the extrapolation factor was multiplied. (π × 0.02352)/(0.0472). Measurements were made using ImageJ software (version 2.0.0) and the MP images were obtained using a high-resolution camera (single-lens reflex digital camera, Nikkon D5600, The Nikon factory, Ayutthaya, Thailand) attached to the microscope [32]. To find non-synthetic sources, a heated needle test was performed [29]. The morphometric traits of MPs including type/shape, color, and sizes were determined by Hossain et al. [32]. Out of 487 possible particles, 15 were chosen for polymer detection. From filter papers, comparatively larger particles (seen 10× magnification under the microscope) were chosen for the Petri dish in order to identify the different types of MP polymers. The polymer type was determined using the potassium bromide (KBr) pellet technique and the Fourier Transform Infrared (FTIR) of an 8400S made by Shimadzu Corporation, Japan (wavenumber range of 4000–400 cm^−1^). For these, 200 mg of KBr powder and 1–3 mg of an MP sample that had been finely crushed were combined and the mixture was then compressed for 1 min under regular pressure of 10 tons in a pellet press, resulting in a clear pellet that was made using a Shimadzu (IR Prestige-21) hydraulic press [33]. The entire system was maintained under evacuation during the preparation of the pellet and this pellet was almost completely analyzed using an FTIR spectrometer with a resolution of 2 cm in 30 scans. The identification procedure involved an automated contrast with the vast spectral databases. By comparing the FTIR spectra with the previously published studies, the false identification relying only on automatic libraries can be eliminated [34,35]. In this case, the IRUG Spectral Database was used. 

### 2.5. Contamination Assessment of MPs in Fish

Environmental danger is frequently measured using the pollutant load index (PLI) in both terrestrial and aquatic environments [36]. In this research, the amount of MPs found in fish from the lower Meghna River estuary was used to determine the environmental risk. PLI at the study location is related to MP concentration factors (CFi). The formulas mentioned below were used to create and categorize the PLI (Table S1) [36,37]. However, no research was conducted to establish baseline readings for MP contamination in the lower Meghna River estuary. Therefore, the background value for the corresponding fish was determined as the minimum concentration of MPs in the DT, gill, and muscle.

### 2.6. Control of Contamination

All liquids such as distilled water and hydrogen peroxide were filtered using cellulose nitrate filter paper with a 5 µm pore size and 47 mm diameter filters. All the laboratory equipment associated with this study were cleaned and rinsed with filtered distilled water before and after use. Necessary precautionary steps were taken to reduce all possible contamination of samples. Moreover, special care was taken throughout the study basically in the time of fish sample collection, transportation, and preservation as well as during the dissection of fish tissue (gill, DT, and muscle). To remove possible contamination by airborne fibers, all the dissecting tissues placed in Petri dishes were covered with aluminum foil paper [29,30]. For a control, one fully blank sample without fish tissue was conducted following the same protocol used to compare the present investigation. No MPs were found in the blank samples [31]. 

### 2.7. Statistical Analysis

Normality and homogeneity of the data were checked before doing descriptive statistics, ANOVA, and Tukey’s test. Correlation and linear regression between microplastic abundance and biological variables were analyzed [38]. The significance level was set at *p* < 0.05 or *p* < 0.01 for each case. All the analyses were performed using the PAST (V. 4.03) software, IBM SPSS statistics (V. 25), and R Studio (v. 3.5.1).

## 3. Results and Discussion

### 3.1. MPs Occurrence and Abundance in Fish

**Figure 1:**
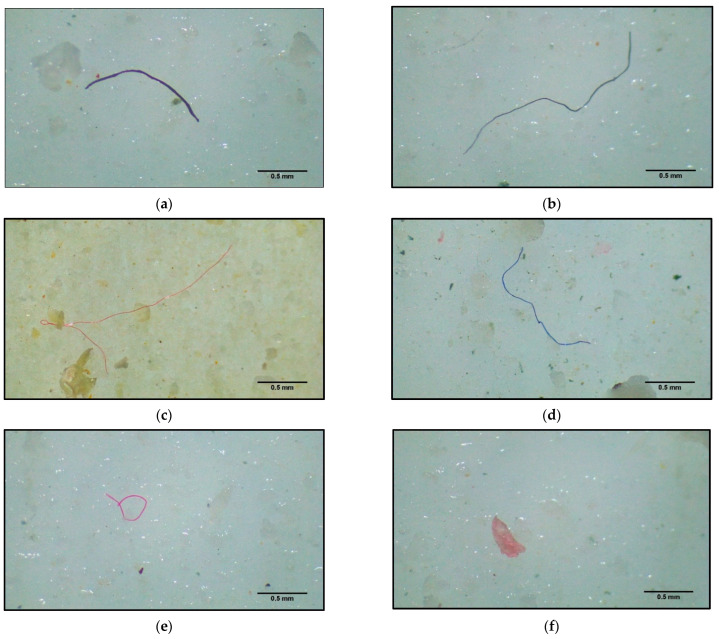
Occurrence of each type of microplastics in the fish examined under a stereomicroscope; (**a**) violet fiber, (**b**) black fiber, (**c**) red fiber, (**d**) blue fiber, (**e**) pink fiber, and (**f**) pink fragment. These particles were found in the gills, gut, and muscle of fish.

**Figure 2:**
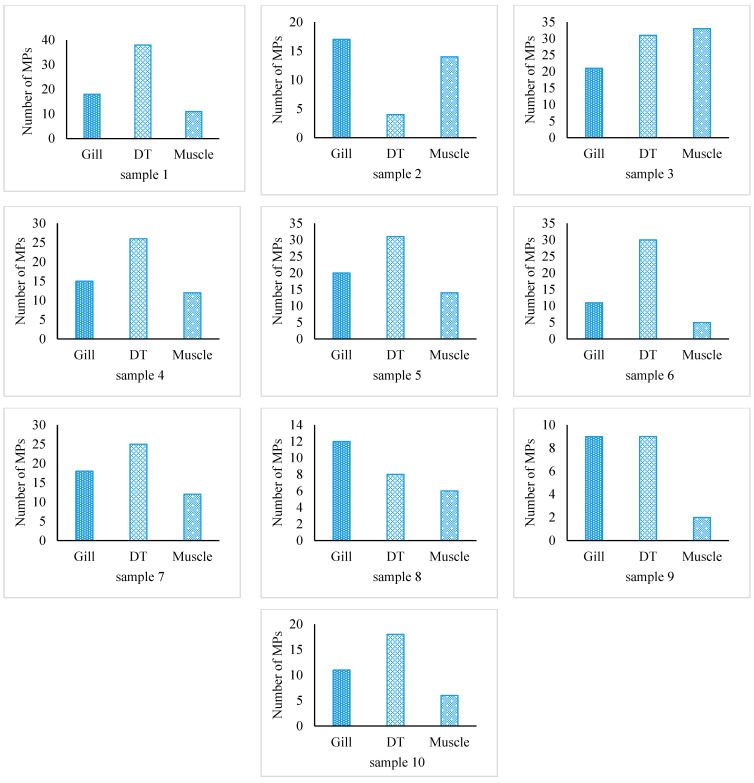
The abundance of MPs for each sample was analyzed by fish gill, muscle, and DT.

**Figure 3:**
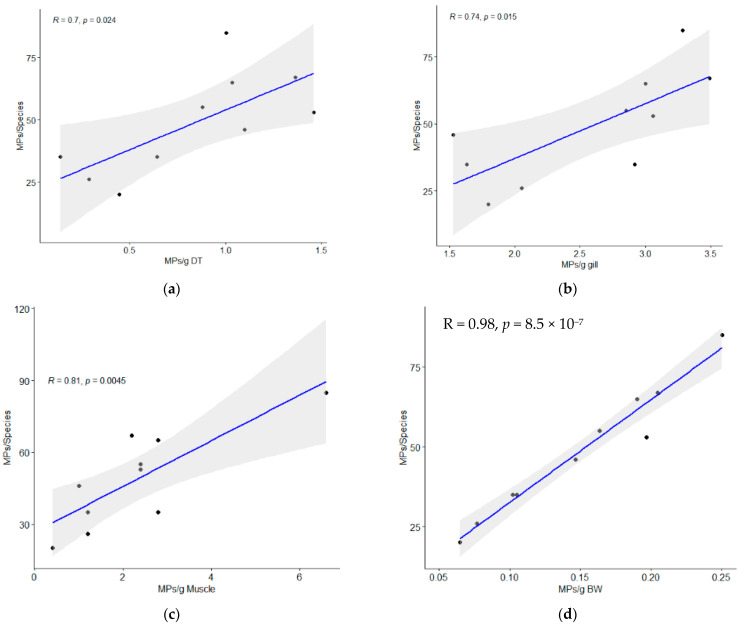
Bivariate plots of extracted MPs per fish species against (**a**) per unit digestive tract (DT) weight (MPs/g DT), (**b**) per unit gill weight (MPs/g gill), (**c**) per unit muscle weight (MPs/g muscle), and (**d**) per unit body weight (BW) (MPs/g BW). Here, solid line = predicted relationship and shaded regions = 95% confidence intervals.

All 10 samples of fish specimens of *S. guttatus* contained MPs (Figure 1) with an average number of 48.7 ± 20.3 MPs/individual (Table 1). In the present study, the total number of microplastics in the gill, digestive tract (DT), and muscle were 152, 220, and 115, respectively. MPs were identified in the digestive tract, gill, and muscle of this species as 0.84 ± 0.45 MPs/g, 2.56 ± 0.73 MPs/g, and 2.3 ± 1.72 MPs/g, respectively (Figure 2). The output of one-way ANOVA demonstrated that the abundance of MPs significantly differed (F = 13.65, *p ≤* 0.0001) between the DT, gill, and muscle of investigated species. The results of Tukey’s pairwise comparisons revealed that MPs/g BW highly significantly differed from MPs/g gill (*p* = 0.0001) and MPs/g muscle (*p* = 0.0002) whereas MPs/g gill did not differ significantly from MPs/g muscle (*p* = 0.9295). In contrast, MPs/g DT significantly differed (*p =* 0.02) from MPs/g muscle (Table 2). Moreover, the amount of MP in fish tissue was positively correlated with the body weight (r = 0.973, *p <* 0.001), muscle weight (r = 0.810, *p <* 0.01), gill weight (r = 0.739, *p <* 0.01), and DT weight (r = 0.701, *p =* 0.05), indicating that fish with a higher body weight will have higher MPs in their gill, DT, and muscle (Figure 3). 

In summary, the data show that when plastics are swallowed, the fish species is harmful, with a maximum of 85 MPs being extracted from a single fish. Studies of fish species that are targeted for commercial purposes have found similar levels of microplastic ingestion in pelagic and demersal species from Turkey’s Mediterranean coast (34%) [39] and benthopelagic fish from Portugal’s western center coast (73%) [40]. The sole previous study in lower Meghna River estuarine waters revealed 100% of individuals ingesting MPs [17]. Nonetheless, ingestion ranges vary greatly among studies, habitats, and sites. The majority of these field studies have generally connected MP ingestion to various fish-feeding techniques [38], vertical dispersion [41], or location, such as closeness to urban or industrial zones [42].

The findings of the present study were compared with the other studies in Table 2. These findings were consistent with microplastic pollution analysis of fish by Hossain et al. [18] and Yagi et al. [43] The MPs concentration/g DT of *S. guttatus* was found to be lower than *Sciades sona*, *Setipinna tenuifilis*, *Priacanthus hamrur*, *Carangoides chrysophrys*, *Otolithoides pama*, *Sardinella brachysoma*, *Harpadon nehereus*, and *Coilia neglecta* fish from the Bay of Bengal [15] and *Zeus faber* fish from the west coast of Kyushu, Japan [43]. In contrast, *Harpadon translucens and Harpadon nehereus* from the Bay of Bengal had lower MP contamination in the DT compared to the present outcomes [15]. 

Previous studies showed that MPs were more common in certain fish organs than in other parts; significant plastic abundance variations between fish’s stomachs and intestines were found as a result of changes to the fish’s weights, structures, and morphologies [41]. However, the amount of plastic pollution in the environment and fish feeding patterns are directly related to the presence of MPs in fish [22]. As a result, when fish feed from the water column or sift through polluted sediment, MPs can be ingested directly (primary ingestion) or indirectly (secondary ingestion via contaminated prey) [44]. In one study, it was discovered that 500–20,000 MPs/km^2^ in the surface waters of the Bay of Bengal could affect the translocation of MPs in fish, which has a discernible effect on coastal plastic pollution [45]. The translocation of MPs from gut to muscle can occur through mechanisms such as absorption through the intestinal walls, entry into the lymphatic system, and distribution via the bloodstream. Factors such as the size and type of microplastics, the fish’s metabolism, and its feeding habits all influence the extent of translocation. Smaller microplastics, such as nanoplastics, may have a higher potential to be absorbed and distributed within the fish’s body due to their small size and ability to pass through cell membranes. The permeability of the fish’s gut plays a role in whether they can cross the gut barrier and enter the bloodstream. When they pass through the gut and enter the bloodstream, they can be distributed throughout the fish’s body via its circulatory system. As blood flows through various tissues, microplastics can become embedded in tissues, including the muscle tissue.

**Table 2:** Comparisons of microplastics (MPs) levels in fish from other tropical areas.

Country	Study Region	Fish Species	No. of Fish	MPs/g DT	MPs/g Gill	MPs/g Muscle	MPs/ind.	References
Bangladesh	Meghna River estuary	*S. guttatus*	10	0.84 ± 0.45	2.56 ± 0.73	2.3 ± 1.72	48.7 ± 20.27	Present study
	Karnafully River	*Setipinna phasa*	30	8.29 ± 1.75	-	-	13.17 ± 0.76	Hossain et al. [46]
		*Polynemus Paradiseus*	30	5.44 ± 0.51	-	-	10.83 ± 0.81	
		*Otolithoides pama*	15	1.65 ± 0.19	-	-	5.93 ± 0.62	
	Bay of Bengal	*Priacanthus hamrur*	10	2.53	-	-	3.8	Ghosh et al. [15]
		*Setipinna tenuifilis*	10	6.45	-	-	3.2	
		*Sciades sona*	10	1.67	-	-	3	
		*Carangoides Chrysophrys*	10	2.5	-	-	2	
		*Sardinella brachysoma*	10	1.82	-	-	2	
		*Harpadon nehereus*	10	1	-	-	1.8	
		*Otolithoides pama*	10	1.2	-	-	1.8	
		*Coilia neglecta*	10	0.94	-	-	1.5	
		*Anodontostoma chacunda*	10	0.45	-	-	1.4	
		*Megalaspis cordyla*	10	0.63	-	-	1	
	Bay of Bengal	Sardinella *gibbose*	25	1.55 ± 0.48	-	-	3.20 ± 1.16	Hossain et al. [18]
		*Harpadon translucens*	25	1.10 ± 0.30	-	-	5.80 ± 1.41	
		Harpadon *nehereus*	25	0.37 ± 0.10	-	-	8.72 ± 1.54	
India	Gulf of Mannar coast	*Sufflamen fraenatus*	20	0.22	-	0.18	-	Selvam et al. [47]
		*Heniochus acuminatus*	25	0.10	-	0.06	-	
		*Pseudotriacanthus*	20	0.35	-	0.36	-	
		*Leiognathus brevirostris*	15	0.12	-	0.10	-	
Japan	West coast of Kyushu	*Scomber japonicus*	40	38	-	-	0.95	Yagi et al. [43]
		Trichiurus *japonicus*	38	17	-	-	0.45	
		*D. tumifrons*	15	1	-	-	0.07	
		*Z. faber*	9	3	-	-	0.33	
		*M. scolopax*	39	3	-	-	0.08	
		*C. equula*	73	11	-	-	0.15	

### 3.2. Morphological Characteristics of Fish MPs

**Figure 4:**
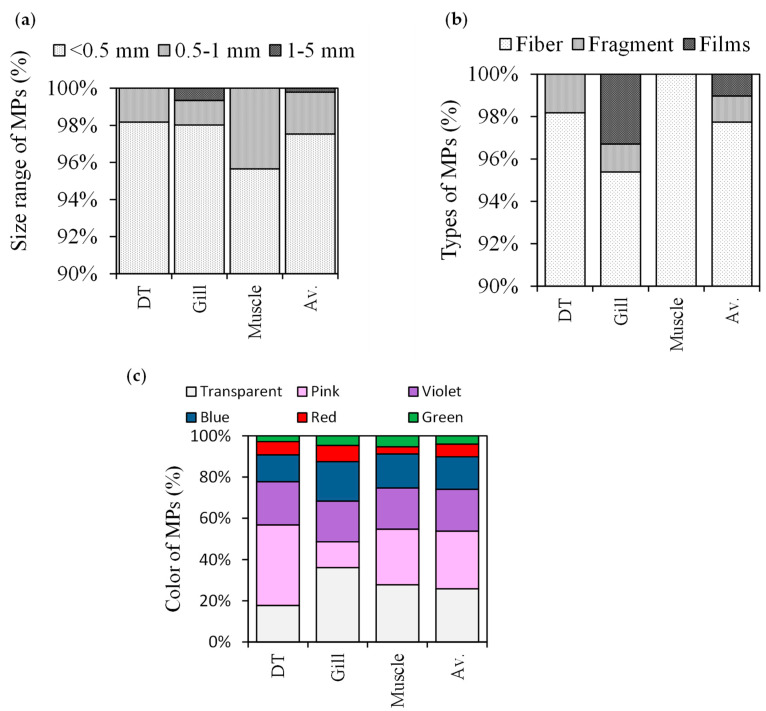
Morphological characteristics of MPs (**a**) size ranges, (**b**) types, and (**c**) color.

All fish samples contained MPs in various shapes, sizes, and colors (Figure 4). The majority of the MPs (97.74 percent of all MPs recorded in this research) were in the 25–500 micron size range, with 0.5 mm and 1 mm accounting for the remaining 5%. In addition, 98.2%, 98%, and 95.7% of the MPs in the DT, gill, and muscle samples, respectively, were smaller than 0.5 mm in size (Figure 4a). A maximum of 0.5 mm to 1 mm (4.3%) was discovered in muscle. The prevalences of the particle classes 0.5 mm and smaller, 0.5–1 mm, and 1–5 mm were 98%, 1.3%, and 0.7% in the gill, respectively. In comparison, DT and muscle did not exhibit any MPs in the 1–5 mm size range. This level is greater than the percentage of small MPs discovered in the DT of fish harvested for commercial purposes from the east Chinese coast and estuaries, which made up 40% of the total items [48]. Since the gills are responsible for performing the functions of respiration, osmoregulation, and excretion as well as providing fish with the ability to filter MP particles from the water [49], relatively larger-sized MPs were detected in the gills in this study. However, the gills are not as well protected as the skin and mouth [50]. According to Eriksen et al. [45], a large percentage of marine plastic debris was composed of fragments smaller than a millimeter, many of which are comparable in size to the tested fish’s natural prey. Similar to this, pelagic and demersal fish in the North Atlantic and the Baltic Sea consumed MPs that were smaller (<0.5 mm) along with their usual prey species [51]. In recent research, the muscles of commercial fish *Serranus scriba* from Tunisian coasts contained MPs that were <100 microns or smaller [52]. The danger of MPs varies depending on their size [53]. We have come to the conclusion that small MPs could pose a significant threat to fish if ingested and that toxicology studies should take note of the negative effects of small microplastics ingested by fish (including bathypelagic fish).

In *S. guttatus*, nearly all of the MPs were fibers of various colors and sizes (Figure 4b). In contrast to the decreasing order of MP types in fish gill, which were fiber (95.4%), films (3.3%), fragment (1.3%) in DT, fiber (98.2%) and fragment (1.8%) kinds of MPs were abundant. Additionally, the muscle of the experimental fish contained only fiber. Fibrous microplastics, however, were common in other investigations as well [15,41,54]. The fragmentation of fishing gear (such as ropes and nets) and recreational sailing gear are the next two main sources of fiber in the marine environment that come from wastewater treatment facilities [55]. MP fibers are more dangerous than other MP particle shapes and prolong the time that fiber accumulates in the gastrointestinal systems [13,56,57].

Transparent, pink-, violet-, blue-, and red-colored MPs were common in the DT, gill, and muscle samples of fish (Figure 4c). The total MPs’ color followed the decreasing order of pink (27.93%), transparent (25.87%), violet (20.33%), blue (15.81%), red (6.16%), and green (3.9%). Transparent MPs were more abundant than microplastics in other colors, contributing for 36.2% of MPs in the gill samples and 27.8% of items found in muscle samples. These proportions were in line with the results observed in fish from Chinese coastal waters [41] and the South China Sea [58]. According to Roch et al. [59], foraging fish were shown to consume MP particles of food-like hues more frequently than non-food-like colors. But the presence of colored MPs suggests that they could be caused by synthetic and organic chemicals, necessitating advanced thorough research. 

### 3.3. Polymer Characteristics of MPs

**Figure 5:**
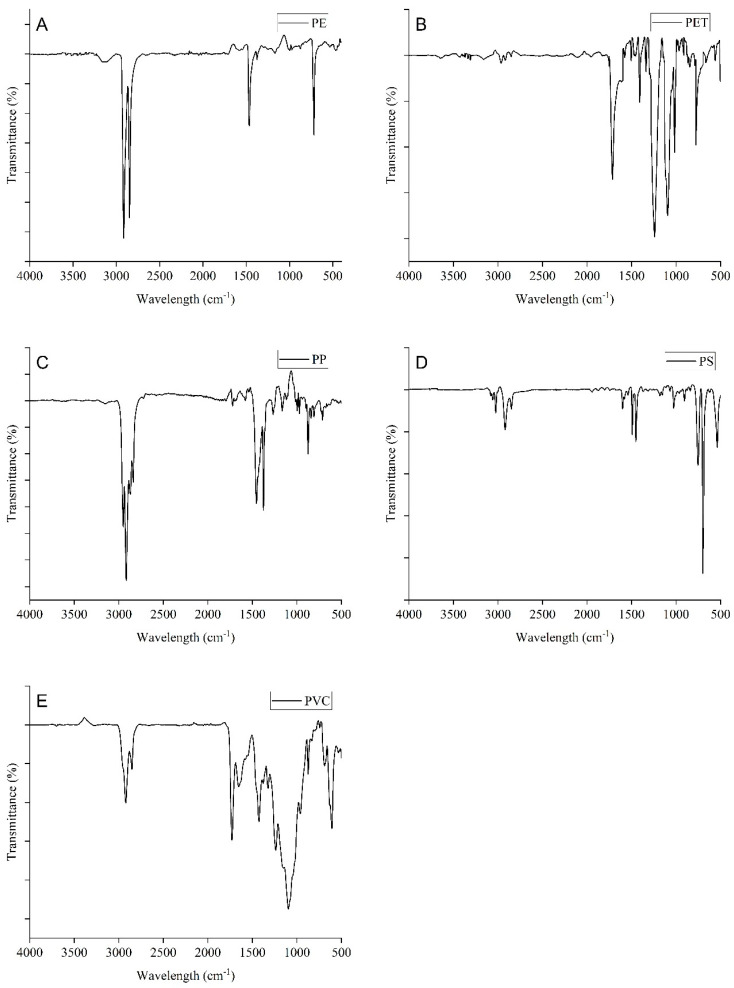
Polymer types identified MPs from the sampled fish; PE (**A**), PET (**B**), PP (**C**), PS (**D**) and PVC (**E**).

MP samples from the *S. guttatus* fish were subjected to FTIR analysis. The findings revealed that PE was the most dominant polymer type (40%), followed by PP (20%), PET (20%), PS (10%), and PVC (10%). The identified polymers of FTIR spectra are showed in Figure 5 along with the respective MP. Previous studies also revealed these types of polymers in riverine ecosystems and different marine fish [15,16,50,60]. Meanwhile, there were some identical picks that were absent in the spectrum of the identified polymers because of the effects of weathering and aging [4]. 

The frequent occurrence of PE in the studied fish might originate from food packaging and the containers of oil, shampoo, soap, and other cosmetic products for leveling [61]. PP is used all around the world for food and beverage packages, plastic containers, and wrapping plastics. Furthermore, the potential sources of PET and PVC are domestic waste and plastic pipes which might be driven to the aquatic systems through river discharge and surface runoff. However, plastic debris deposited by tourists and locals also increases the load of plastic in the sediment of the marine environment. On the other side, the main potential source of PS is fishing activities in the river which are used by fishermen usually to extrude polystyrene (XPS) and expanded polystyrene (EPS) as buoyant. Moreover, further research is suggested to assess the point sources of MPs to prevent MP contamination in the aquatic ecosystem.

### 3.4. Contamination Level Assessment 

The CF values of fish muscle (5.75) and the digestive tract (5.50) indicated that these fish organs were considerably contaminated (3 < CF < 6) with MPs whereas gill (1.69) had moderate contamination levels (1 < CF < 3). PLI is calculated to quantity the degree of MP pollution [16,62]. The PLI values of the fish organ samples were >1, indicating the contaminated condition of *S. guttatus* fish. The PLI values followed the decreasing order of the muscle (4.53), digestive tract (4.52), and gill (1.63). As PLI was calculated using the ratio of MP occurrence to background value, the polymer type of MPs appears to have no impact on PLI [63]. However, human activities such as industrialization, fishing, population density, water transportation, etc., are what cause MPs to occur in seawater [16,64].

## 4. Conclusions

This study highlighted the ubiquitous presence of MPs in a commonly consumed fish, *S. guttatus,* from a large subtropical estuary in Bangladesh for the first time. MPs were found with an average abundance 48.7 ± 20.27 item/individual which was higher compared with species from other estuarine environments. The abundance of MPs in gill was significantly higher than that in the DT and muscles due to the continuous contact with water and first reaction against any unfavorable conditions in an aquatic environment. Furthermore, MPs’ abundance was positively related to the body weight, DT weight, and gill weight of the pelagic fish indicating that fish with a higher weight will have higher MPs in their gill, DT, and muscles. The majority of the particles were of the fibrous type, transparent and pink in color, and generated primarily from synthetic origin; the breakdown and degradation of fishing equipment (plastic lines, ropes, and nets) as well as smaller size (<0.5 mm) plastics were prevalent in DT, inferring that MPs could be actively uptaken by fish because of their similarity with natural foods or by assuming plastics as prey or through trophic transfer microplastics. Different types of polymers such as PP, PE, and PET were isolated from the MPs. High PLI values (>1) indicated significant commination levels. Hence, the possibility of human exposure to microplastics through ingestion raises concerns owing to the probable transfer of smaller-sized MPs and hazardous contaminants into edible tissues. 

## Figures and Tables

**Table 1:** Fish morphometry and their corresponding levels of MP ingestion.

Scheme.	TL (Range) cm	BW (Range) g	DT Weight (Range) g	Gill Weight (Range) g	MPs/g DT	MPs/g Gill	MPs/g Muscle	MPs/g BW	MPs/ind.
*S. guttatus*	36.68 ± 1.53 (33.9–38.2)	325.22 ± 22.8 (269.3–342.8)	26.74 ± 4.26 (17.8–30.93)	6.0 ± 0.79 (4.9–7.2)	0.84 ± 0.45	2.56 ± 0.73	2.3 ± 1.72	0.15 ± 0.06	48.7 ± 20.27

## Data Availability

Data are contained within the article and supplementary materials.

## Supplementary Materials

The following supporting information can be downloaded at https://www.mdpi.com/article/10.3390/biology12111422/s1.
