# Supplementary material for: Microplastics in Different Tissues of a Commonly Consumed Fish, Scomberomorus guttatus, from a Large Subtropical Estuary: Accumulation, Characterization, and Contamination Assessment
Source: Biology (Basel). 2023 Nov 12;12(11):1422. doi: 10.3390/biology12111422 (PMC10669230; doi:10.3390/biology12111422)
Supplement: Supplementary file 1 [file biology-12-01422-s001.zip › biology-2655305-supplementary.pdf]

Table S1: Contamination level assessment indices

Eq.	Index	Depiction and objectives	Principle	Explanation	Pollution degree criteria
1.	Contamination factor (CF)	CF is used to estimate the MPs contamination.	$CF = \frac{C_{(Sample)}}{C_{(background)}}$	Here, $C_{(Sample)}$ is the concentration of MPs, $C_{(background)}$ is the background concentration of MPs.	CF<1: Low contamination<br>1<CF<3: Moderate contamination<br>3<CF<6: Considerable contamination<br>CF>6: Very high pollution
2.	Degree of contamination (CD)	CD is used to evaluate MPs	$CD = \sum CF$	Here, CF is the contamination factor which is determined by Eq. 1.	CD < 5: low<br>5<CD<10: moderate<br>10<CD<20: considerable<br>20>CD: very high
3.	Pollution Load Index (PLI)	PLI can be used for assessing of comparison of contamination status among the study sites.	PLI=$\sqrt[n]{CF1 \times CF2 \times CF3 \times \ldots \times CFn}$	Here, n is the total number of samples, and CF is calculated as described in the earlier equation (Eq. 1).	PLI<1: No pollution;<br>PLI>1: Polluted
